# A genetic journey through cancer: from rarity and family to aspirin and nanowires

**DOI:** 10.1186/1897-4287-10-S2-A30

**Published:** 2012-04-12

**Authors:** J Burn

**Affiliations:** 1 FMedSci, Newcastle University, UK

## 

Rare diseases illuminate the most important molecular steps along cell pathways. A clinical encounter with a woman crippled by the autosomal dominant turban tumour syndrome or cylindromatosis led us to chromosome 16 and the CYLD gene, later shown to be a key regulator of ubiqitination in the NFkappa B pathway and a potential target for high dose salicylate therapy. A genome wide approach added dysregulated tropomyosin kinase signaling (Trk).We developed an *in vitro* cylindroma primary cell culture model which permitted experimental validation of Trk inhibitors as a viable therapeutic approach.

Familial studies in our centre have focused on hereditary cancer. Two large pedigrees led to collaboration with Kolodner in identification of the mismatch repair pathway and rapid entry into diagnostics which, in turn, led to development in 1994 of our international CAPP2 randomised controlled trial of aspirin and resistant starch in 1009 carriers of an MMR gene defect. We have demonstrated, in prolonged double blind follow up that taking 2 aspirins daily for 2 years results in a subsequent 60% reduction in new cancers at a mean of 5 years (paper in press). COX2 inhibition is an unlikely sole mechanism for such delayed effects. Enhanced apoptosis, similar to that seen in infected plants triggered by natural salicylates, would explain the effect. Another under-explored mechanism open to modulation is the immune response to mismatch repair deficient cells. Lymphocytic infiltration is the hallmark of these tumours and recent studies by our research partners have revealed striking levels of immune response to the frame-shift derived neopeptides released by MMR deficient cells. These antibody responses provide a credible biomarker for our planned CAPP3 dose inferiority study which will examine different doses of aspirin in 3000 gene carriers. This immunological discovery also opens the way to possibility of a vaccine against the neopeptides which would have relevance for the 1 in 6 sporadic cancers which manifest acquired MMR defects.

Simpler methods to investigate tumour genomes in the pathology lab are needed because while aspirin can be offered to all, most new therapies call for greater targeting. . Our commercial venture, QuantuMDx hopes to fill this gap. Tethering single strand DNA to a silicon nanowire and sequencing with heavily charged chain terminating nucleotides in a microfluidic vehicle offers robust cheap genotyping “while you wait”, converting chemistry to computer code without the “middlemen”. Affordable and reliable testing for microsatellite instability and activating ras mutations could then be easily incorporated into standard histopathology practice. Proof of principle is anticipated within months and a commercial device is planned within three years.

If the method proves to be as robust as we anticipate, it offers cheap genotyping in pharmacies for common variants such as the UGT1A6 polymorphisms which modulate response to aspirin and would allow more personalised dosing as we move towards a recommendation of general aspirin use in the over 50’s for prevention of vascular events and cancer.

